# Myth or reality? Some directions on translation universals in recent corpus based case studies

**DOI:** 10.3389/fpsyg.2022.902400

**Published:** 2022-11-16

**Authors:** Juan Jia, Muhammad Afzaal, Swaleha Bano Naqvi

**Affiliations:** ^1^School of Foreign Languages, Zhejiang University City College, Hangzhou, Zhejiang, China; ^2^Institute of Corpus Studies and Applications, Shanghai International Studies University, Shanghai, China; ^3^NUST Business School, National University of Sciences and Technology (NUST), Islamabad, Islamabad, Pakistan

**Keywords:** translation comparison, translation universals, corpus-based studies, translation and interpreting, machine translations

## Abstract

This paper contributes to the ongoing debate on the existence of translation universals (TUs) by mapping the theoretical literature on the TUs and evaluating selected corpus-based studies which investigate a swath of hypothesized universals. Based on a review of empirical research carried out over recent years in translation studies, this paper attempts to develop a holistic picture of the evidence on TUs pertaining to multiple aspects of translation. We found that although some evidence for certain hypothesized universals exists, it cannot be definitively claimed that TUs comprise an indisputable reality. Based on review of the studies surveyed, the present study concluded that several universal claims within research, over the period of time, proved to have been falsified at a universal level, while being proved right as lower-level translation modes or determined generalizations for particular types. Thus as the hypotheses regarding universals have been worked upon on a very small range of languages or pair of languages, it is imprudent to declare them as translation universals.

## Introduction

In view of the growing disciplinary interface of translation studies with newer methodologies, a key question which arises pertains to the applicability of corpus-based research to translation universals. In essence, this invites speculation over whether translators and scholars of translation studies should treat the existence of translation universals (TIs) as a type of distinct computational task. We argue that there is an urgent need to inquire into translation universals as these are possibly drawn upon by translators to simplify translations for greater audience accessibility. The trajectories of questions described constitute attempts to rethink the concept of translation universals.

One of the foci of the present study was to review the phenomenon of translation universals which have been widely explored and discussed and even misunderstood to some extent in the field of translation studies. In particular, scholars of Translation Studies (TS) hwave tended to focus on developing theories in corpus-based translation studies or identifying translation universals in translated or non-translated corpora (e.g., Baker, 1993; Laviosa, 2009; Chesterman, 2010a,b), thus extending the debate on the concept of TUs in Translation Studies. This review begins by mapping the theories, concepts and laws proposed by numerous translation studies scholars. In this context, we discuss Baker’s conceptualization of translation universals, Toury’s laws as well as Chesterman’s theories and newly developed concepts which support or challenge the existence of translation universals. The review continues with an examination of four recently published case studies through a quantitative analysis of translated corpora. Chesterman (2004a,b) distinguishes between different types of translation universals in the following way. While an S-universal relates to “universal differences between translations and their source texts” (Chesterman, 2004a,b, p: 39), a T-universal refers to “the typical differences between translations and non-translations in the target language” (Chesterman, 2010a,b, p: 40).

Essentially a T-universal makes a claim about something that is typically different between translations and non-translations in the target language, whereas an S-universal makes a generalization about a difference that exists between source texts and translations. It is important to keep in mind that both types are concerned with differences in relation to the reference texts they use. Nobody has pointed out that the fact all translations tend to be equivalent in some way to their source texts or that all translations count as texts in the target language in themselves serve as interesting translation universals. Neither of these claims has been made. Pym (2008), on the other hand, has put forward yet another possible explanation for some universals. For instance, in his discussion of Toury’s two laws [e.g., the laws of (i) increasing standardization and (ii) interference from the source text], Pym argues that both can be explained by translators’ desire to minimize the possibility of error. The law of standardization “refers to the tendency of translators to adapt foreign features of the

source text to the cultural and linguistic inventory in the target culture” (Tully, 2014, p: 295), whereas the law of interference pertains to “an opposite and contradictory tendency to transmit the foreignness of the source text into the target culture” (Tully, 2014, p: 295).

With the onset of the twentieth century, the emergence of digital technology (corpus-based translation studies) gave rise to considerable methodological developments and stretched the canvas of translation thinking and translation research (Schulte, 2020). These newly introduced computer tools made it possible for the development of critical literature on the re-translation of literary texts, while foregrounding the so-called recurrent questions or concepts prevailing in the field of translation studies. These questions have tended to range from interrogating the rationale for retranslating certain texts, probing the difference between retranslations and first translations and examining the datedness of translations in contrast to the non-datedness of original texts (Massardier-Kenney, 2015).

The phenomenon of translation universals has been questioned on several counts. For instance, it is argued by Chesterman (2004a,b, p: 42) that even if such universal features are discovered in translations, robust testing and control of variables would be needed to ensure meaningful conclusions. In the study of linguistics, a language universal is an attribute that is asserted to be universal and to be present in every language. Hypotheses regarding language universals can be tested against a significant portion of the languages spoken around the world. On the other hand, when it comes to translation universals, the situation is different, as the sum of all translations that have ever been carried out in the world in the past and in the present is of an extremely different magnitude. A second caveat offered by Mauranen (2008, p: 35) probes the very nature of the universality of the TU hypotheses (e.g., instantiation without exception in all translations versus the extent of TU occurrence in all types of translated texts). If it is assumed that universality pertains to the likelihood of instantiation in all types of translations, Tymoczko (1998, p: 5) cautions that as corpora tend to be founded on Western archetypes, they cannot truly represent all translated texts across the dimensions of languages, location and time. Tymoczko’s argument is that this would mean running the risk of hypothesizing universals on the basis of a very narrow sample.

There is also concern voiced by Pym (2008) that the four TUs proposed by Baker (1996), despite their apparent diversity, actually represent aspects of the same universal. For instance, explicitation, normalization/conservatism, implification as well as leveling out act to improve the readability of the translations in the target culture. A fourth criticism in relation to TUs is that they appear to be contingent upon conditions within the target culture, with translators evidencing a tendency to seek standardization (or adoption of target culture features) if the translations lack importance and status within the target culture. Due to the above, claims made about “universals” need to be interpreted in a more nuanced manner within the context of translation research. For this reason, some academics prefer to use other terms, such as general tendencies or patterns, or even just generalizations, provided that they are qualified and conditioned as appropriate.

## Existing studies on translation universals

The concept of translation universals appears to have evolved within empirical translation-based research. The phenomenon has become a core concern for translators and scholars of translation studies (Toury, 1995). This section explores the recent developments and theoretical insights from studies conducted on the basic trigonometry of so-called translation universals and the features of these universals in the field of translation studies. The debate on translation universals starts is incomplete without a discussion of Baker’s work (1993, 1995, 1999, 2004) and her contribution to the identification of so-called translation universals and presentation of corpus tools in translation studies. However, despite the current expansion of translation studies and the shift from manual translation to machine translation as well the integration of artificial intelligence, the concept of translation universals is poorly understood. In this regard, De Sutter and Lefer (2020, p: 7) point out that “fundamental questions [as to] which social, pragmatic and cognitive mechanisms shape translation, how these mechanisms interact, and to what extent this interaction functions differently than in other types of monolingual and bilingual written language production” remain unresolved. It would appear that the scholars who are interested in translation universals find it difficult to distinguish between translated and non-translated texts as these tend to be the products of hypothesized, rather than unanimously-agreed upon, translation universals presented in disparate studies (De Sutter et al., 2012, p: 326).

Over the passage of time, Translation Studies has drawn the attention of researchers such as Károly (2007) who showed an interest in the identification of general features characterizing any translational text. Although earlier researchers like Levý (1965) repudiated the term “translation universal,” this did not curb his interest in studying the linguistic features evident in translations. Levý was led to conclude that analytical as well as rational methods of evaluation were needed to identify the features effectively. House (2008, p: 11) negates the existence of translation universals by arguing that “translation is an act that operates on language so any behaviour observed in the translation process is a behaviour that applies to all language use.” In other words, the translational language is lexically, syntactically, and stylistically simplified in comparison with the source language (Tsai, 2020). A key criticism of the translation universals hypothesis was that Baker’s study of the Translational English Corpus (TEC, Laviosa, 1997, 1998) was confined to English as target or source language and disregarded the origins of the author, genre, and source language (Martin, 2017; Tsai, 2020).

The study by Baker (1993) became the foundation for the understanding of the phenomenon of translation universals. Baker advanced the field of translation studies by advocating for a shift in orientation from source text to target text and an accompanying shift from the normative concept of ‘equivalence’ to the descriptive concept of ‘norms’ (as cited in Sutter and Lefer 2020). Baker observed that these were principles that needed to be taken into account in order to understand translation behavior when working with the differences between the original texts and their translations. Baker considered them to be universal owing to the fact that these phenomena are unavoidable because of the constraints that are inherently present in the process of translation (1993, p: 246).

Supporting Baker’s concept of translation universals, De Sutter and Lefer (2020) point out that despite the fact that Baker’s schema was exploratory and not meant to be used as a theoretical framework, it has been erroneously deployed as a framework by many researchers, thus giving rise to much (conceptual) ambiguity over TIs.

Drawing upon Baker (1993) study, Laviosa-Braithwaite (1998; evaluating the general features of any translation) carried out an independent corpus analysis and generated a definition for the characteristics typical of translations published in the Encyclopedia entry of Translation Studies (1998). Universals of translation were delineated as linguistic features which typically occur in translated rather than original texts and are thought to be independent of the influence of the specific language pairs involved in the process of translation (Laviosa, 1998, p: 288). However Baker (1996) notion of levelling out which pertains to the proclivity of the translated text to be drawn towards the continuum midpoint has been left out by Laviosa (2009).

The universals identified by Laviosa are based on the ideas presented by Baker (1993) which range from simplification, explicitation to normalisation (conservatism according to Baker). To this, Laviosa adds the avoidance of repetitions present in the source text as well as Toury (1995) law of interference (i.e., discourse transfer, and the idiosyncratic distribution of lexical items of the target-language). The 2009 edition of the Routledge Encyclopedia of Translation Studies maps the progress and changes that have been made in translation studies since Baker’s work (1993). Drawing upon the ideas presented by Toury (2004) and Chesterman (2004a,b), Laviosa (2009, p: 306–311) observes that the value of the general laws characterizing translated texts lies in their explanatory power which allows them to clarify unique phenomena. Toury (1995) law of interference among the universals and the law of growing standardization, in addition to the description of the classification given by Chesterman (2004a,b) as an endeavor to classify the translation universals are elaborated upon by Laviosa (2009, p: 306–311). Chesterman notes that translation universals should be categorized on the basis of how they become known when they are put in equivalence against the source texts or when a comparison is made with the characteristics of the original texts. When compared to benchmark texts, both approaches highlight the differences.

## Different conceptualizations of translation universals

We reviewed different concepts because it is important to contrast the ideas to examine indexical theories of translation universals. Asserting that universality requires a linguistic phenomenon to occur only in translation texts and in no other text, Pym (2010, p: 78) argues that these linguistic universals should be amongst the classifications for the characteristics of translated texts. On the other hand, empirical research results (e.g., Puurtinen, 2004; Saldanha, 2004; Becher, 2011) point to the non-existence of such inherent features in all translations, thus compelling the researchers to argue that the phenomenon revealed cannot be relevant to all types of texts and to the contexts of their translations (Tymoczko, 1998).

A possible solution to the conundrum of TIs lies in probabilistic translation laws and their establishment (Chesterman, 1993, p: 3). In a later publication, Chesterman, (2010a,b) suggests that term “universal” should be used in its “weaker” meaning and sense when the general features of the translations are taken into consideration which warrants a focus on equivalents, namely the statistical universals of the absolute universals. Furthermore, when discussing the translations and the characteristics that are observable, it is suggested that the universal tendencies of translated texts should be considered. With respect to explicitation in the context of translation universals, Dimitrova (2005, p: 40) observes that although TIs have the potential to instantiate universally, it is not at all necessary that they appear in every case of translation. Research by key scholars of translation studies has yielded a number or premises, including an account of S-universals and T- universals. The following table presents a summarization of hypotheses by multiple scholars (Source: Chesterman, 2004a,b):

With reference to the hypothesis that translated texts tend to be lengthier than the source texts. Vinay and Darbelnet (1958, p: 185) note that this is not always true and is likely to be contingent upon the languages involved and their linguistic features.

The two ‘laws’ of translation offered by Toury (1995, p: 267–279) include the ‘law of growing standardisation’ and the ‘law of interference’. While the first law observes that ‘in translation, source text textmes tend to be converted into target text repertoremes’ (Toury, 1995, p: 268), the second law asserts that ‘in translation, phenomena pertaining to the make- up of the source text tend to be transferred to the target text’ (Toury, 1995, p: 275). To explain, the law of standardization suggests that translators have a tendency to replace textual relations within source texts (e.g., unique collocation) with relations which are more inclined towards norms in the target language (Palumbo, 2009, p: 69–70). In the case of the law of interference, what this suggests is that linguistic features within the source text language tend to transfer to translated texts, at times resulting in negative transfer (departure from standardized target language practice; Palumbo, 2009, p: 69–70). With reference to the idea of dialect normalization, this implies that ‘translations tend to normalize dialects’ [Dimitrova (2005) in Manca (2016), p: 146].

Originating in the journal Palimpsestes and within essays by Berman and Bensimon in particular, the re-translation hypothesis holds that re-translations tend to be closer to the source text than the first translations (Mihálycsa and Wawrzycka, 2020, p: 2). Blum-Kulka first came up with the idea of explicitation in 1986 (Bednář, 2015, p: 3). She defined it as comprising “cohesive explicitness from SL to TL texts regardless

of the increase traceable to differences between the two linguistic and textual

systems involved” (Blum-Kulka, 1986, p: 300). The process implied adding ‘semantic, syntactic or lexical elements to elucidate information and relations which are more implicit in the source text’ (Bednář, 2015, p: 3). In contrast, the process of implicitation comprised ‘making implicit information which was clearly and explicitly stated in the source text’ (Bednář, 2015, p: 3).

Explicitation may be understood as a ‘phenomenon whereby a translated text is seen to convey information in a more explicit form than in the original text’ through use of connectors and explanations to unpack culturally-mediated terms in the source text (Palumbo, 2009, p: 47). While explicitation may arise due to strategic choices made by the translator, it may also arise due to tendencies integral to the text which has been translated (Palumbo, 2009, p: 47). Mauranen (2008, p: 39) advises that the idea of explicitation must be viewed with caution, as it is possible that other variables (e.g., temporal/cultural gaps between ST and TT languages) may influence the shaping of the translated texts. The notion of explicitation revolves around the premise that translations are inclined towards being more explicit that their source texts (Blum-Kulka, 1986), a notion widely explored in many studies (e.g., Klaudy, 1996; Dimitrova, 2005). Despite being studied widely, the concept has been elucidated in divergent ways which makes comparing of the results (e.g., Becher, 2010) all the more challenging.

The discussion of potential T-universals starts with the description of simplification. Simplification may be understood as a “frequently hypothesised translation universal [involving], among others, breaking up long sentences in the process of translation” (Laviosa, 2002 in Kajzer-Wietrzny et al., 2016, p: 235). In break-through research based on a comparison of translated texts (fiction and newspaper) to source texts in terms of lexical density, core lexis and sentence length amongst other measures, Laviosa (1998) noted these to constitute key aspects of simplification. Untypical lexical patterning may be understood as “lexical patterning which differs from that which is found in original target language texts (thus comprising) a universal feature in the language of translations” (Mauranen and Olohan, 2000, p: 136).

## The escalation of universals in translation studies

Translation universals have been variously conceptualized, with understandings of this phenomenon ranging from the view that it comprises translation behavior and may be understood as a law, thus implying the possibility of there being exceptions to the law (Toury, 1995), represents transfer operations (Klaudy, 2004) and characteristics of linguistic phenomenon (Pym, 2008) to cognitive phenomena (Baker, 1996). Chesterman’s (2004a,b) efforts to connect S-universals and T-universals did not prove fruitful due the complexity and ambiguity of the hypotheses.

In the period following the 1970s, translations took center stage as a separate text type, namely “a third code which arises out of the bilateral consideration of the matrix and target codes [and]is, in a sense, a sub-code of each of the codes involved” (Frawley, 1984, p: 168). Toury suggested that the “laws” of translation should be treated as descriptive generalizations (1995 and earlier versions). Translations, as one law stated, showed a manifestation of interference from the original text while another pointed out that they leaned towards a standardized version, more so than the original. These ideas have made the whole process of translations more interesting and have begun to drive the need of the researchers to find out the underlying reasons for these phenomena. Studying how translators translate texts and what constraints they are facing, cultural or otherwise, can help to develop an understanding of how they are able to work with different languages.

Mona Baker’s initiative of bringing methodologies of corpus studies at Manchester paved the way or researchers to come up with and test the new claims, to generate translations and its features (Baker, 1993; Laviosa-Braithwaite, 1998) which were earlier considered to be typical. Corpora of similar nature became the norm as the data for comparable corpus began to increase. Based on the model of the linguistic search, since Baker (1993), “translation universals” have become a norm which the scholars term as potential generalizations for language universals.

Despite many issues discussed further on, “universal” has become a widely used term when dealing with translation corpus studies. There are two different kinds of “universals” in the form of an S-universal and a T-universal (Chesterman, 2004a,b). While both types of universals highlight the differences as compared to their reference texts, the source texts and their translations are not considered equivalent and the translations are not counted as texts in the language they have been translated into. Some of the more notable universals claimed or highlighted as hypotheses (from Chesterman, 2004a,b) are discussed below. These include the under-representation of target-language-specific items. The “unique items hypothesis” put forth by Tirkkonen-Condit (2004) for the first time, has begun to be studied widely (see Chesterman, 2004a). This hypothesis suggests that target-language items which are noticeably different from a particular source language (and in this sense “unique”) will not be utilized very often in the translation owing to the fact that they will most possibly be readily available in the repertoire of the translators. This is based on the supposition that the form of the source language is responsible for the mental processing (for example, translations into Finnish will have fewer-than expected particles like -pA or -kin, if there is a lack of similar particles in the source languages).

Another assumption that arises is the existence of a cognitive cause which might elucidate how the translators are able to process texts in two languages simultaneously. Halverson’s hypothesis of gravitational pull (Halverson, 2003, 2007) has been one of the most interesting propositions, which talks about the pull on the process of decision making because of the target-language prototypical or highly salient forms. These noteworthy forms emerge in a translator’s mind which lead to some of the T-universals, e.g., simplification. Likewise, there may also be a pull from the forms of the source texts which would lead to interference. Halverson expounds on these effects of the pulls by placing them in the realm of cognitive grammar.

Another potential explanation for some of the universals has been put forth by Pym (2008). While discussing two of Toury’s laws, he argues that translators follow these universals to avoid any kind of risk. For example, literal translations (which might involve interference) are a way to play safe, especially if there is ambiguity of any sort in reaching a conclusion to the exact meaning of the source text. The use of high-frequency forms can also be seen to be cautious not only to avoid the use of unnatural language which happens when the translator is not translating a text into his first language but also to ensure that the message is sent across to larger and wider readership. Other factors that might come into play can be the constraints of deadline might which might affect the processing of thoughts and which might lead to decisions concerning choices to be made for safe and quick solutions. Contrastive rhetoric and analysis can also be used to some extent to explain S-universals. The different that is found in style and language between the target and source language can also account for some generalizations that cover the translation between a pair of given languages (e.g., the supposition that translations are longer that the source texts), further making them more “universal” with the increase in the number of differences found.

## Ongoing criticism on the existence of TUs

Research on universals has attracted significant criticism, primarily pertaining to terminology. While the hypotheses of the presence of language universals can always be tested against the large number of languages in the world, the presence of translation “universals” is entirely a different case altogether. This is why “universals” in translation research have to be considered in a weaker sense, with researchers being inclined, as discussed earlier, to talk about general tendencies or patterns, or simple generalizations which can be taken as necessary conditions.

A related flaw has been pointed out by Tymoczko (1998), amongst others. Whenever the need arises to generate or test a hypothesis regarding a universal, a corpus of translations is built. However, it is not clear as to what should or should not be counted as a translation in order to be included in the said corpus. For example, we need to answer a few tricky questions to decide this monumental step which include asking if the translations should be included if they are done by native speakers of the target language and if they are recently published. Questions may also pertain to whether they can be categorized as good or as bad or if they are executed by trained professionals, amateurs, groups, fans or individuals and comprise adaptations or versions. When constructing such a corpus, it needs to be clear where to draw a line at including items.

Some critics have also noted that the manner in which universal assertions are expressed and operationalized is not always clear to the reader. An excellent illustration of this is the nebulous concept of explicitation. The first version of Blum-suggestion, Kulka’s which was published in 1986, focused on the distribution of markers of coherence; nevertheless, the idea of explicitation in general has been interpreted and classed in such a variety of ways since then that it is impossible to make any broad conclusions.

Those who believe that research on universals is largely futile base their criticism on the fact that it only tends to highlight elements of translations that are already pretty well known—as features of poor translations. The foregoing discussion refers to derogatory statements typically made about translations. According to such perspectives, the new corpus studies only demonstrate that these derogatory views are correct and that such studies do not bring anything new to the discussion on translations.

## Re-interpreting translation universals

Despite extensive criticism leveled at translation universals, considerable research on TUs has been conducted within the ambit of corpus-based translation investigations. The translation universals have been reinterpreted to elucidate the interrelationship between apparently contradictory perceptions predominant in earlier theories.

According to Halverson (2007), the general characteristics of the human cognitive process seem relevant to the translation universals in terms of their features. However, according to Frawley’s (1984, p.168) theorizations, translation in its essence comprises ‘a sub-code of each of the codes [source and target codes). Frawley also suggests that translation may be viewed as two opposing gravitational forces. In this regard, Frawley has compared the pull of two opposite forces to translational processes, classifying these as target language pull or the unusual distribution of firm target language elements attributed to interference (i.e., source language pull).

**Figure 1:**
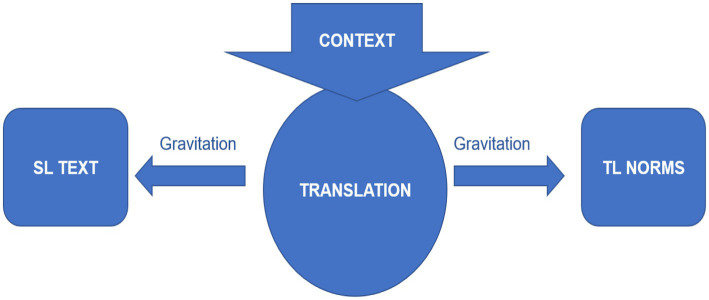
The translation process at the intersection of two attracting gravitational forces. Figure reproduced from Halverson (2003), with permission from John Benjamins Publishing.

**Figure 2:**
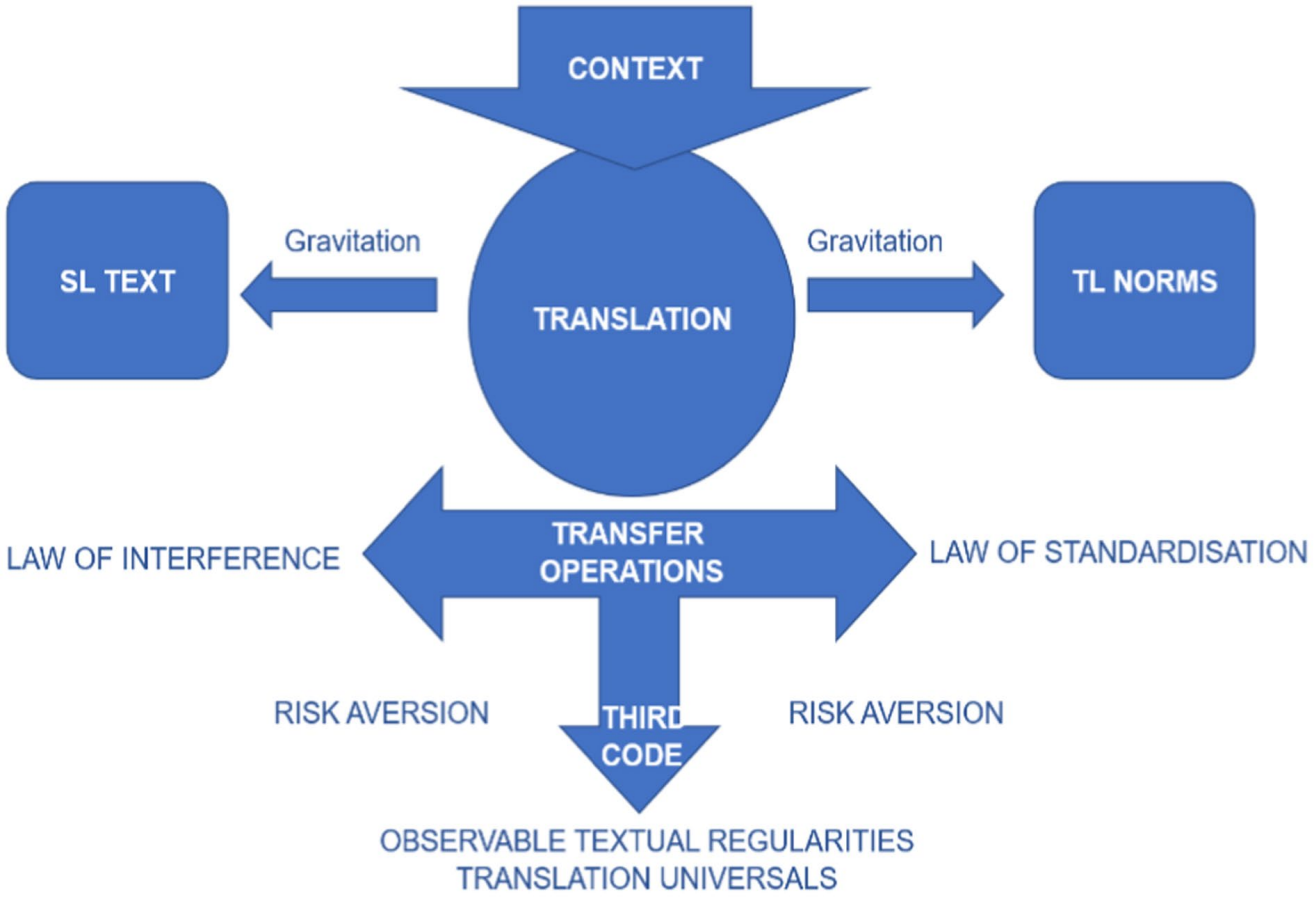
The model of translation universals’ inception. Figure reproduced from Robin (2017), with permission from John Benjamins Publishing.

Based on Halverson’s (2003) hypothesis, contextual factors may influence the gravitational pull as demonstrated in Figure 1 shows the process of translation in terms of gravitational forces, and the interference of contextual factors in the process of translation from source text to target text. However, the real scenario relates the two forces to Toury’s theory of laws of translation stating the laws of growing standardisation and interference. On the other hand, Halverson (2003) also discusses the element of growing standardisation based on the idiosyncratic features of the ST which usually gets modified during the process of translation. While the law of interference states that translators usually adopt all features of ST to TT during the process of translation, Halverson (2003) terms this interference negative due to the transfer of characteristics of ST structure to TT, while negating the rules of TL. Only one consequence of interference is accepted, namely if the transfer or interference is likely to crop up in the translation due to features of ST which happens due to the gravitational pull (Halverson, 2003). Drawing upon Halverson’s (2003) model of translation processes, Robin (2017) proposes an expanded model of the translation universals focusing on law of interference and law of standardization (Figure 2).

Figure 1 shows the process of translation in terms of gravitational forces, and the interreference of contextual factors in the process of translation from source text to target text. However, the real scenario relates the two forces to Toury’s theory of laws of translation stating the laws of growing standardisation and interreference. On the other hand, Halverson (2003) also discusses the element of growing standardisation based on the idiosyncratic features of the ST which usually gets modified during the process of translation. While the law of interreference states that translators usually adopt all features of ST to TT during the process of translation, Halverson (2003) calls this interference negative due to the transfer of characteristics of ST structure to TT while negating the rules of TL. Only one consequence of interreference is accepted, namely if the transfer or interference is likely to crop up in the translation due to features of ST which happens due to the gravitational pull (Halverson, 2003).

**Table 1:** Potential S-universals and T-universals. Based on data from Chesterman (2004a,b, p: 8).

**Potential S-universals**
Lengthening: It talks about the length of translation that should be longer than source texts (Vinay and Darbelnet, 1958, p: 185)
Toury’s introduction to the law of standardization (1995)
Toury (1995) the law of interference
Dialect normalization by Dimitrova in 1997
Taivalkoski-Shilov (2002) reduction of complex narrative voices
The concept of the explicitation hypothesis by Blum-Kulka (1986)
Kenny (1998) sanitization
Palimpsestes’ (1990) re-translation hypothesis
Baker (1993) reduction of repetition
**Potential T-universals**
The concept of simplification by Laviosa-Braithwaite (1998) stating less lexical variety, lower lexical density, and more use of high-frequency items
Baker (1993) conventionalization
Mauranen and Olohan (2000) untypical lexical patterning
(Chesterman, 2004a,b, p: 8)

In addition, Toury (1995) has talked about the contextual factors in the process of translation and enhanced the concept with two more factors, i.e., socio-cultural and extralinguistic factors. Toury (1995, p: 22) states that:

the more peripheral translations are in the target language culture, the more the translated texts endeavour to conform to the general, established practice of the system of the target language, i.e., the law of standardisation does not exert its influence on the translation process and thus on the produced text in all cases or with the same intensity.

Therefore, the debate on gravitational pull, contextual factors and some others extralinguistic factors reflect constraints inherent in translation as stated by Baker (1993), and linguistic factors which are used in the production of translation text (Károly, 2007). In the meanwhile, such a process of translation is further modified into a new model of translation universals highlighting the universal textual features of translations which can be analyzed with the use of scientific calculations. Therefore, the process of translation universals has been presented while keeping in mind the linguistic phenomenon in the following figure.

House (2008) argues that translation universals mediate the translation process and the operations involved in the creation of translated text led to translation universals. During the creation of translated text, there can be manipulation of ST and the optional operations entails explicitations and implicitations (Weissbrod, 1992, p: 155), implying that their use is influenced by all the factors that have an impact on the implementation of the laws guiding them. Pym (2008) considers motivation as the key issue that manifests risk aversion. Chesterman (2004a,b) defines these risk-aversions as “text level shifts (S-universals) and drifts as T-universals which are outlined in Table 1.

The following section provides a critical review of selected case studies examining the instantiation of translation universals in various aspects of translation research.

Focusing on machine translation, the study by Luo and Li (2022) examines whether the translated texts produced with the help of WeChat Translate, a MT tool provided by a popular social media app in China, evidence TUs typical of human translations. Luo and Li investigated whether two hypothesized translation universals in particular (simplification and normalization) transpire in WeChat Chinese to English translated texts. While simplification suggests that translators are prone to “simplify[ing] the language or message or both” (Baker, 1996, p: 176), normalization is a term used to describe the inclination of the translators to “conform to patterns and practices which are typical of the target language, even to the point of exaggerating them” (Baker, 1996, p: 176). In the event of the examined TUs transpiring in the data, the researchers also sought to inquire into the linguistic patterns of the feature as well as the underlying reason for it. The study found that while the simplification TU could not be confirmed through analysis of the corpus under study, a tendency towards normalization was identifiable. Luo and Li suggest that this was likely to be due to the way in which machine translation operates swiftly, without the need for the laborious effort required by human translators which inclines the latter towards simplification in the first place. Lu and Li also found that, given the MT systems’ reliance on reference corpora based on standard language and translation paradigms, WeChat was primed by key words in source texts to repetitively select normative grammatical choices in the targeted language.

The phenomenon of translation universals has also been evaluated in a recent study conducted by De Sutter and Marie (2020). De Sutter and Marie focused on the critical evaluation of corpus-based translation studies, highlighting limitations, recent developments and new methodologies adopted these days in the field of translation studies. The subsequent focus of this paper is on the recurrent hypotheses within translation studies on the existence of translation universals. De Sutter and Marie (2020) hypothesize that “activeness will appear as part of interaction effects, not as main effects.” This hypothesis is based on the work of Wulff et al. (2018) which built a large comparable corpus of native and nonnative student writing. This comparable corpus comprised almost 274,000+ and 164,000+ tokens, taking 198 essays from Dutch component of International Corpus Learner English (ICLE).

The study identifies the general difference between explicit and implicit in the writing of native and non-native learners. De Sutter and Marie (2020) identified that the preference for explicit signaling by Dutch learners is much stronger than that evidenced in the writings of English peers. The results indicate clause boundary as 74% out of 363, with the percentage for Dutch learners of English approximating 81% and English native showing 68%. The comparison of native and non-native writing is shown in the following figure taken from De Sutter and Marie’s study (2020). De Sutter and Marie (2020) compared variables in linear mixed-effects model, presenting the main effect of nativeness in the following figure extracted directly from their work.

This study reveals that there is a statistical significance in nativeness length, MC Verb, and CC subject without significant multicollinearity or overdispersion issues (De Sutter and Marie (2020), p: 17). They further reveal that their model provided 89% accuracy score in the results and identified that native writers tended to write more implicitly than L2 learners. Therefore, we selected another case study conducted in 2020 by Yvonne Tsai titled “Diachronic observation of lexical and syntactic patterns in English translations of Taiwan patent texts” which is discussed in the following section.

The study by Yvonne Tsai (2020) identified features of patent translations with the particular domain of lexical density type-token ratio (TTR) to investigate the simplification in terms of average sentence length. The issue of measuring length and complexity has been under the core agenda of many research studies (Laviosa-Braithwaite, 1998; Magalhaes and Batista, 2002; Xiao and Yue, 2009; Azimi et al., 2012), as simplification in translated texts leads to simpler lexical, syntactic and stylistic features (Blum-Kulka, 1983).

An earlier study by Tsai (2010) showed that TTR in translated text remains lower in English writings, signifying that translated texts contains less lexical variation in a comparison to non-translated text. The results presented in his study revealed that English translated text showed lower TTR than that evidenced by non-translated texts. In addition, the lexical variations in translated English texts were found to be lower than non-translated English texts. While analyzing her results, Laviosa (1998) argues that translated text remain less varied and less lexically dense than non- translated text. This anticipates the findings of Tsai (2020) recent study which suggests that simplification is evidenced in translated texts. Blum-Kulka’s hypothesis on explicitation suggested that translators demonstrate cohesive markers in TT which are not found in ST. Blum-Kulka (1986) provided evidence from various texts. Baker (1996, p: 180) described this issue as “the tendency in translations to ‘spell things out rather than leave them implicit’ “. When we analyse a language on the basis of lexical variation and syntactic approach, the phenomenon of explicitation can be assessed through the identification of parts of speech used in translation or written texts (e.g., conjunctions, adverbs, or the use of relative pronouns). Tsai’s recent study (2020) argues that relative pronouns are most frequently occurring pronouns in texts, leading to greater explicitation in translation.

Tsai (2020) analyzed the use of ‘which’ and ‘that’ across different periods in non-translated texts. In the 1995–2000 period, a high frequency of ‘which’ was found, but this dropped dramatically to the least used relative pronoun in the 2001–2006 period. In comparison to translated texts, his study showed more frequent use of ‘which’ and ‘that’ in 2013–2018. Pastor et al. (2008) applied a corpus-based NLP approach to examine the TUs of convergence and simplification within Spanish translations. The comparable corpora were selected from medical and technical domains, and within the former the translated texts were created by trained translators as well as by students. Based on comparative analysis of the selected corpora, Pastor et al. found that although simplification does influence translations, the translations created by untrained translators do not provide evidence of simplification. Their study did not find evidence for the instantiation of convergence, particularly in regard to differences of syntax between the corpora under study.

Zasyekin’s study (2016) focused on a psycholinguistic approach to examine translation universals for modeling literary creative translation. This research identified a common psycholinguistic technique for translating fictional writings from English into Ukrainian and studies translators’ universal strategies based on the psycholinguistic model of literary translation and experimental evidence demonstrating its validity (Zasiekin, 2016, p: 22). This study was also reinforced by an empirical psycholinguistic examination of translation S-universals that comprised procedural and discursive regularities, thus enabling a description of the translator’s cognitive/analytical and synthetic resources. They entail intuition and associative thinking, which mentalists and connectionists have described convincingly. Zasiekin (2016) also revealed that the discursive S-universals continue to hold their status as common methods utilized by translators regardless of the cognitive approach taken by the translators. In contrast, the TAP analysis of 34 protocols revealed that the procedural S-universals were mostly impacted by the dominant channel of source text perception held by the translators. This was the case for all of the procedural S-universals.

In another study of translation universals, de la Fuente and Fuertes (2015) investigated the oral production of bilingual children. The concepts of simplification and explicitation, two of the most well studied universals in the field of translation research, are the focus of this investigation. They investigated the oral production of bilingual children using a variety of language pairs that are available through the CHILDES project and focus on two main questions: the first is whether or not instances of simplification and explicitation appear in the production of non-instructed interpreters, and if they do, how their occurrence relates to the type of data (i.e., spontaneous or experimental) and the language pair that is being interpreted. The second question is whether or not instances of simplification and explicitation appear in the production of instructed interpreters. According to the findings, children who are learning two first languages frequently translate between the languages, in addition to employing simplification and explicitation to varying degrees depending on the language combination. de la Fuente and Fuertes (2015) concluded that the examination of acquisition data has the potential to help illuminate the characteristics of these translation universals.

The critical review of the studies discussed above suggests that the evidence on translation universals is divergent, with some studies finding limited evidence for specific hypothesized universals and others finding little validity of other TUs. For instance, Luo and Li’s investigation, while not finding support for simplification, found that the translations reflected normalization, possibly due to the fact that WeChat relied on reference corpora based on standard language norms. Other research like the one carried out by De Sutter and Marie (2020) revealed a tendency on the part of native writers to write more implicitly than non-native writers. Limited evidence for explicitation and simplification in translation research has been suggested, respectively, by Tsai (2010), Pastor et al. (2008) and Tsai (2020).

Researchers working on translation universals have failed to demonstrate that the differences they have observed between non-translated and translated texts are, in fact, caused by the hypothesized translation universal rather than by something else, as pointed out by De Sutter et al. (2012, p: 326). According to Halverson (2003), the Gravitational Pull hypothesis was proposed in an attempt to explain certain differences in human cognition by referring to the characteristics of human cognition in general. A number of patterns proposed as being unique to translation are most likely natural effects of bilingual language production rather than universal characteristics of the translation process, according to the author (Halverson, 2013). Using the argument that translation is an act that operates on language, House (2008, p: 11) asserts that translation universals cannot exist because any behaviour observed during the translation process is a behaviour that applies to all language use.

## Conclusion

This article mapped the background on the concept of translation universals and reviewed selected studies investigating hypothesized universals within the domain of translation research. The paper began by elaborating on the theoretical concepts provided in support of or against the existence of translation universals. Then it moved onto exploring the phenomenon by analyzing findings from selected case studies focusing on translated and non-translated texts with a view to probing the existence of translation universals. Further, this paper identified how research has shown several universal claims, over the period of time, to have been falsified at a universal level, while being proved right as lower-level translation modes or determined generalizations for particular types. The challenge lies in the fact that as the hypotheses regarding universals have been worked upon on a very small range of languages or pair of languages, it is imprudent to declare them as translation universals. Critics have also pointed out that the way in which translation hypotheses have been formulated and operationalized is not entirely clear, thus making it difficult for researchers to prove or disprove them.

A way out of this conundrum over translation universals has been suggested by Chesterman (2014, p: 87) who argues that a better approach is to regulate the scope of the hypotheses and seek conditioned generalizations at a lower stratum of generalizability that take into account specific languages, profiles of translators, working circumstances and genres. As Chesterman points out, this would allow translation researchers to test the conditioned hypotheses and develop more meaningful information such as how individual translation features relate and the governing contextual conditions. In turn, this would lead to the generation of broader hypotheses targeting the identification of correlations and cause and effect relations. The stance taken by Chesterman (2014, p: 88–89) is premised on the idea that given the fact that translations are mediated by culture, a pluralism of perspectives and approaches are to be expected. Nonetheless, this does not mean that any attempts to seek an identification of common features (as in TUs) must be sacrificed at the altar of a “naïve relativism” which acknowledges only differences in translations Chesterman (2014, p: 89). Adopting this approach would mean not only that all parts of a framework that seeks to describe and explain translation broadly must be subjected to testing but also that the hypothesis being tested must be aimed at deepening understanding or addressing a problem in the field Chesterman (2014, p: 89).

While this study sought to probe the myth or reality of translation universals by examining empirical research in the topic area, a limitation of the current research was that only a selected number of studies conducted in recent years were surveyed. In future research, including a larger number of studies and taking a diachronic approach to the sampling of the empirical studies on TUs may result in richer and more conclusive findings.

Given the inconclusive support for translation universals in the studies surveyed within this paper, drawing upon some robust directions offered by researchers Taghavi and Hashemi (2021), we encourage future researchers to direct their attention, amongst other trajectories and foci, to:

- More corpus investigations of Translation Universals to gauge the applicability of the proposed TUs (hitherto largely based on Western languages) in non-European languages.
- Investigation of whether the hypothesized TUs always manifest through similar linguistic mechanisms/choices within all languages. For instance, simplification always occurs through reductions in lexical density and/or syntactic complexity.
- Research on a range of genres within translated texts to ascertain whether the TUs instantiate in every text type.
- Examination of the impact of influential variables such as translator skills and background on translation behavior with reference to TUs.

## Author contributions

All authors listed have made a substantial, direct, and intellectual contribution to the work and approved it for publication.

## Funding

This research is supported by Scientific Research Foundation of Zhejiang University City College (No. X–202221).

## Conflict of interest

The authors declare that the research was conducted in the absence of any commercial or financial relationships that could be construed as a potential conflict of interest.

## Publisher’s note

All claims expressed in this article are solely those of the authors and do not necessarily represent those of their affiliated organizations, or those of the publisher, the editors and the reviewers. Any product that may be evaluated in this article, or claim that may be made by its manufacturer, is not guaranteed or endorsed by the publisher.
